# Understanding the relationship between care team perceptions about CHWs and CHW integration within a US health system, a qualitative descriptive multiple embedded case study

**DOI:** 10.1186/s12913-022-08723-7

**Published:** 2022-12-27

**Authors:** Erin E. McCarville, Molly A. Martin, Preethi Lakshmi Pratap, Eve Pinsker, Steven M. Seweryn, Karen E. Peters

**Affiliations:** 1 University of Illinois at Chicago, School of Public Health, 1603 W Taylor Street, Chicago, IL 60612 USA; 2 University of Illinois at Chicago, Institute for Health Research and Policy, 1747 West Roosevelt Road, Chicago, IL 60608 USA

**Keywords:** Community health worker, Integration, Healthcare, Purpose, Value, Role

## Abstract

**Background:**

Community health workers (CHW) have grown in prominence within the healthcare sector, yet there is no clear consensus regarding a CHW’s role, purpose, and value within health systems. This lack of consensus has the potential to affect how CHWs are perceived, utilized, and ultimately integrated within the healthcare sector. This research examines clinical care teams that currently employ CHWs to (1) understand how members of the care team perceive CHWs’ purpose and value, and (2) consider how perceptions of CHWs are related to CHW integration within health care teams.

**Methods:**

Researchers conducted a qualitative descriptive multiple embedded case study at the University of Illinois at Chicago’s Hospital and Health Science System (UI Health). The embedded subunits of analysis were teams within UI Health that are currently employing CHWs to assist with the provision of clinical care or services to patients. Data were collected via semi-structured interviews and document review.

**Results:**

In total, 6 sub-units were enrolled to participate, and 17 interviews were conducted with CHWs (*n* = 9), and administrators or health care providers (*n* = 8). Reported perceptions of CHWs were inconsistent across respondents. CHWs roles were not always understood, and the CHW’s purpose and value was perceived differently by different members of the care team. Moreover, evaluation metrics did not always capture CHWs’ value to the health care system. In some cases, care teams were more aligned around a shared understanding of the CHW’s roles and purpose within the care team. When perceptions regarding CHWs were both positive and aligned, respondents reported higher levels of integration within the healthcare system.

**Conclusions:**

Alignment in a care team’s perception of a CHW’s role, purpose, and value within the health system could play an important role in the integration of CHWs within healthcare teams.

**Supplementary Information:**

The online version contains supplementary material available at 10.1186/s12913-022-08723-7.

## Background

CHWs have a long history of community-based health service delivery, but recent trends have contributed to the popularization of CHW models within health and hospital systems [15, 18]. Some in the sector, recognizing this movement, argue that CHWs are an “emerging healthcare workforce” in the US [27], or that “CHWs are poised to enter the mainstream of healthcare [1].” But “without careful and thoughtful consideration, CHWs could get lost in the healthcare system [1].” The question of how to effectively integrate CHWs into a healthcare system is critical.

One notable challenge is the potential for differences in care philosophy and approach between a traditional healthcare workforce and CHWs. CHWs and healthcare providers may operate with different underlying paradigms related to disease prevention and health promotion. While CHWs often view their work to be long-term and relationship-driven, traditional healthcare models are more commonly transactional and time-limited [11, 29]. In healthcare, health problems are “solved” with treatment. Whereas CHWs “understand” health problems within the greater environmental context; and it is the process of understanding the contextual factors associated with a problem that enables CHW’s to help patients improve their health [16]. Inclusion of CHW programs within the healthcare context requires a careful consideration of how to integrate these different approaches and philosophies.

Additionally, CHWs do not typically gain expertise through traditional healthcare training or certification channels. It is therefore difficult for those in the healthcare sector to easily understand what roles or services CHWs can provide [18].

CHW roles tend to be broad and varied depending on the needs of the communities served [6, 16]. While national standards for the CHW workforce have been established [5], inconsistency in CHW roles remains on the local programmatic level. Additionally, some individuals within the healthcare sector may not understand the purpose or value of a CHW workforce. Without a clear consensus regarding a CHW’s role or purpose, it remains difficult to integrate CHWs into healthcare teams.

This research strives to examine programs that are currently employing CHWs in the provision of clinical care to (1) understand how different members of the care team perceive a CHW’s purpose and value, and (2) consider the role of CHW perceptions in CHW integration.

## Methods

### Study design

This study is an exploratory case study which utilized cross-case comparison among clinical teams as sub-units of analysis using interview and document review data. The case of study is the University of Illinois at Chicago’s Hospital and Health Science System (UI Health). Teams within the UI Health System that employ CHWs to assist with the provision of clinical care or services to patients were recruited as embedded subunits of analysis. UI Health is an academic hospital system based in the near west side of Chicago. Part of the University of Illinois at Chicago (UIC) system, UI Health includes a 465-bed tertiary care hospital, 21 outpatient clinics, and 11 federally qualified health center locations.

**Fig. 1:**
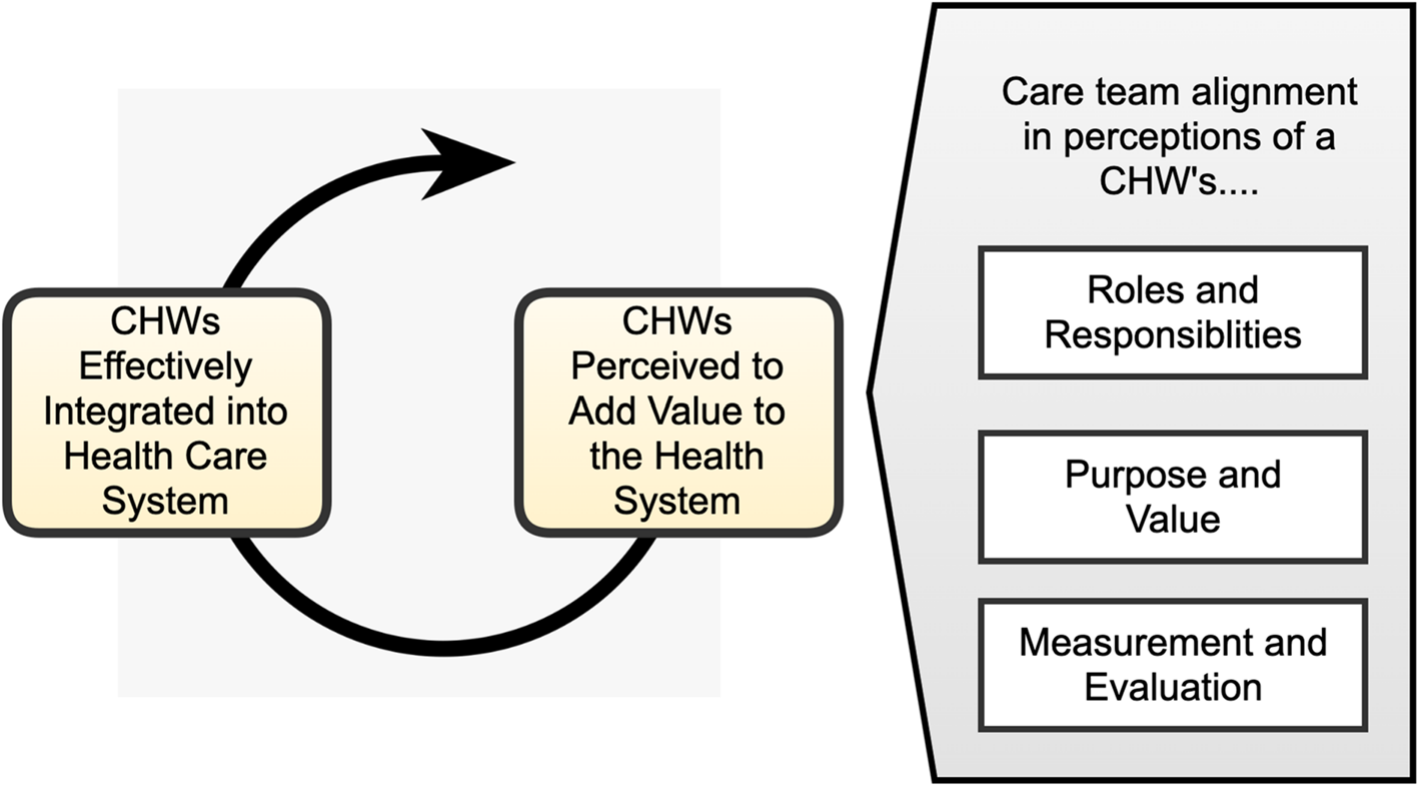
Conceputal framework for CHW integration within healthcare teams

Consistent with best practice in case study research [34], an environmental scanning process (key informant interviews and literature review) was employed to develop a conceptual framework for CHW integration (Fig. 1) [3, 8, 10, 14, 17, 21, 24, 25, 31]. This conceptual framework theorizes that perceptions about CHW’s value to a health system are influenced by a CHW’s roles and responsibilities, a CHW’s purpose within the healthcare team, and how CHWs are evaluated, and that these factors are moderated by the extent to which there is clarity in expectations of the CHW workforce. The research questions, interview guide, and coding scheme were organized to evaluate this theory. Table 1 provides definitions for constructs in the framework and links these to the a priori codes and interview questions used for this component of the research study.

**Table 1:** Definitions of constructs, a priori codes and related interview questions for the research

Construct	Connection to the literature	A priori sub-codes	Code definition	Related interview questions
Roles and Responsibilities^a^	A description of the roles performed by CHWs. This may include formal job descriptions or informal descriptions of the tasks and/or responsibilities of CHWs. A literature review published by Hartzler et al. found that CHW roles in clinical settings could be categorized into three primary categories: (1) clinical services, (2) community resource connections, and (3) health education and coaching. (Hartzler 2018) But the IMPaCT model notably adds an additional CHW role—creative social support—which is not always included in all CHW models. (Kangovi 2018)	Clear definition	The extent to which a CHW’s role is clearly defined and articulated. This may include whether or not different members of the care team share a common definition regarding a CHW’s role.	Can you please describe your current role at UIC? a. What is your official job title? Do you have a different informal title? b. What are your primary roles and responsibilities? c. What department do you work in?
		Clinical services	“Examples include assessment of vital signs, lifestyle, health knowledge, psychosocial factors, and care through routine exams aided by remote communication with physicians. These services provide for patient dialog, helping care teams understand patients’ health, background, and preferences.” (Hartzler 2018)	
		Community resource connections	“Community resource connections link patients with community-based services, such as referrals for transportation or food assistance” (Hartzler 2018)	
		Health Education and Coaching	“Health coaching generally involved motivational interviewing and action planning to help patients achieve health goals. Health education typically targeted specific issues, such as cancer screening or self-management of a chronic illness.” (Hartzler 2018)	
		Social Support	The ability to tailor interventions to patients needs which may have little to do with medical care. This may involve leveraging family, community, or grass-roots resources. It also may include the engagement of CHWs with the patient in the process of intervention or service delivery. (Kangovi 2018)	
Purpose and Value	The stated purpose for hiring/employing a specific group of CHWs. The purpose may be different among different members of the program or clinical care team. Some view CHWs as a strategy to address what has been identified as the “Triple Aim” in healthcare—improved patient experience, health of populations, and reduced cost. (Berwick, 2008) While others view CHW services through a health equity lens. Value involves how individuals define a CHW’s specific unique skills or qualities. The IMPaCT program at Penn defines CHW’s unique value as (1) they represent the populations that are being served, and (2) that they are “natural helpers.” (Kanvovi 2018)	Health Equity	Health equity is defined by the WHO as “the absence of avoidable, unfair, or remediable differences among groups of people, whether those groups are defined socially, economically, demographically or geographically or by other means of stratification.” (who.int)	*For CHWs:* What do you view your purpose to be in your job? What do you think your patients/clients value most about you? What do you think the people that you work with at UIC value most about you? In what ways do you contribute to the work being done by clinicians at UIC? In what ways do you contribute to the health of the patients/clients that you work with? What are your own personal goals in your work? How successful are you in meeting your goals? *For administrators:* Why are you working with CHWs? What are the primary jobs that the CHW performs? Why are you working with CHWs? What are the primary jobs that the CHW performs? What do you value most about CHWs? [*probe: what problems do CHWs help to solve?*] What do you think patients/clients value most about CHWs? How do CHWs support (or not support) the goals of your program/department?
		Reduced Cost	Defined by the IHI as “reducing the per capita costs of care for populations” refers to the goal of achieving a measurable decrease in the cost of the provision of health care to target individuals or populations. But reduced costs may also refer to reduced costs for organizations, groups or individuals within the health system (such as a hospital system).	
		Health Outcomes	Defined by the IHI as “improving the health of populations,” health outcomes refer to a measurable reduction in disease morbidity and/or mortality for groups or populations of people.	
		Patient Experience	Defined by the IHI as “improving the individual experience of care” patient experience refers to how patients feel about their health care experience including people, places and processes.	
		Community Representatives	The extent to which CHWs share similar characteristics—such as race, ethnicity, language, country of origin, income, education and/or community of residence with the populations that they are serving.	
		Natural Helpers	The extent to which CHWs meet the definition of a “natural helper. A natural helper is defined as individual who is “innately empathetic and altruistic.” This may be demonstrated through listening skills, emotional intelligence, or a history of helping and caring for others.	
Measurement	The metrics used to measure or evaluate CHW performance or effectiveness. This may include quantitative or qualitative metrics. It also may include metrics related to patient outcomes or improvements to systems or workflows. The Institute for Clinical and Economic Review (2013) released a guidance paper regarding measurement of CHW outcomes. The following categories of measurement were included: process, knowledge/behavior change, satisfaction, health outcomes, or costs. (ICER 2013)	Process Measures	A consideration of the CHW process. This may include adherence to specific targets (number served, number of visits) or it may include assessments of improvements to processes, workflows or patient access.	*For CHWs:* How do you track whether your patients have made progress? *For administrators:* What are your goals/objectives for CHWs? When you think of CHWs why are they important to your work? How do you (or your department) evaluate CHWs? From your perspective, are CHWs meeting your goals/objectives?
		Behavior Change	The extent to which an individual patient changes a behavior that has been linked to improved health outcomes. For example, smoking cessation, change in salt intake, or change in prescription adherence.	
		Satisfaction	A consideration of how satisfied individuals are with CHW services. This may include assessments of patient satisfaction or it may include satisfaction among other care providers due to improvements in work flow or patient care.	
		Health Measures	The use of health metrics that measure an individual patient’s health status. This may include measures of disease control (such as CD4 or A1C), health status (blood pressure, cholesterol), or health management (hospitalizations, ER visits)	
		Costs/ROI	The use of metrics to measure the costs associated with healthcare services. This may include an assessment of Return on Investment (ROI) or a consideration of insurance incentive payments.	

^a^Each construct is represented in the conceptual framework. Constructs were also a priori code

### Sample selection

All care teams identified as employing CHWs within UI Health were recruited to participate and assessed for eligibility. Care teams were contacted via email for recruitment if they self-identified as employing CHWs in a previously conducted internal organizational CHW survey, if they listed CHWs in reports, websites or publications (identified via internet search), if they were part of existing organizational or regional CHW communities of practice or advocacy groups, or if they were referred by other known CHW programs or CHW experts. The recruitment list was shared with a stakeholder group of CHW experts for review to ensure completeness.

Subunit recruitment was initiated first by assessing eligibility and obtaining approval from the individual or group with management authority over the subunit (e.g., administrator, director, or principal investigator). To be eligible, the care team must self-identify as employing CHWs to assist with the provision of care or services to patients. During recruitment, participants were provided with a formal definition of CHW to ensure alignment in inclusion of CHW groups [2]. Once eligibility was confirmed and leadership approval was obtained, researchers recruited up to 5 participants per sub-unit representing (1) CHWs (*n* = 1–3 per subunit), (2) administrators (*n* = 1 per subunit), and (3) clinicians (*n* = 1 per subunit) when applicable. Participants were recruited over email or phone call. Subunit documents associated with CHW programming or services were also collected for review.

### Measures and measurement

A semi-structured interview guide, developed for this research, was designed for a 60-minute interview (see interview guide). Interview questions included CHW roles and responsibilities, perceptions of a CHW’s purpose and value, and metrics for evaluating CHW performance (Table 1). A document review matrix was also created to collect document data including program information and structure (e.g., number of CHWs, size of caseload, program budget).

### Data collection

Program administrators and/or research participants were invited to share documents including: (1) CHW training documents (manuals, agendas, or evaluation instruments); (2) CHW job descriptions; (3) clinical or CHW protocols; (4) reports prepared for funders or outside agencies; (5) publications; and (6) other relevant documents describing the CHW program. An online search was also conducted to identify publicly available documents including websites, program reports, or publications.

Next, individual one-on-one semi-structured interviews were conducted with 1–4 representatives from each sub-unit over video chat (Zoom) and audio recorded. Interviews were conducted by the principal investigator (EM), a doctoral student in public health. While the principal investigator was employed by UIC in community health work, they did not supervise or work with any of the research participants and they introduced themself as a doctoral student. The interview guide was pilot tested prior to use. When appropriate, the semi-structure interview guide was modified in response to data collected in the document review phase to eliminate redundancy or add clarifying questions. Memos were written at the end of each interview capturing initial researcher thoughts regarding overarching themes or key impressions*.* All study procedures were approved by the University of Illinois at Chicago Institutional Review Board (protocol #2020–0326).

### Data analysis

Data were analyzed by the Principal Investigator (EM) first on the sub-unit level, beginning with document review and followed by interviews. Document data were summarized in Microsoft Excel and document-specific memos were written. Interview recordings were transcribed verbatim, edited to ensure accuracy, and de-identified. Interview data were analyzed used MaxQDA software (version # 2018.2, VERBI Software) and thematic coding. Researchers applied a hybrid coding approach beginning with a priori codes derived from the literature [22]. In a subsequent pass, emergent codes were also developed utilizing a more inductive, grounded approach to identify new or previously unrecognized patterns [30]. An independent coder reviewed a subset of interviews and coders met to review and discuss the coding scheme. This cycle was repeated until a minimum of 80% cross-coder agreement was achieved.

Documents and interviews from each subunit were triangulated within subunits and codes were analyzed across data sources (interviews and documents) to identify points of convergence and divergence [4]. Memos were written to generate a list of subunit-level themes. This was repeated until thematic saturation was achieved [28]. A report summarizing themes was prepared for each subunit and shared with research participants from the respective subunit for validation via a member checking process [34]. During member checking, research participants were invited to respond to a brief survey indicating whether the report accurately reflected their sub-unit and whether they had and corrections or edits to suggest. No respondents had concerns with or edits to the subunit reports.

Themes were then triangulated across subunits to identify convergent and divergent patterns through the charting method [9]. Discussions, reflection, and the resulting memos helped identify cross-subunit themes, thus unifying concepts and interrelationships across subunit data.

## Results

Of 9 identified eligible programs, 6 subunits were enrolled in the research study (66% of eligible programs). 3 declined due to insufficient time or inactive CHWs. Between 1 and 4 interviews were completed for each subunit for a total of 17 interviews (9 CHWs and 8 clinicians/administrators). Mean interview duration was 46 minutes (range = 23–62 minutes). 34 documents were reviewed including 4 job descriptions, 13 reports/publications, 4 websites, 12 training documents, and 1 protocol.

### Perceptions

#### Roles and responsibilities

Respondents were asked to describe a CHW’s roles and responsibilities. There was diversity in how CHWs were employed in the provision of services within the health system. This could be observed in the broad range of CHW job titles, service populations, service delivery models, and roles and responsibilities (Table 2). Different formal job titles were used for CHWs both within and across sub-units. Some programs also assigned “informal” job titles that were distinct from the formal human resource title. Due to specific hiring limitations within the organization of study, none of the respondents reported a formal job title of “Community Health Worker”, but identified informally as such. The CHW’s target population or focus area was also different across subunits—some CHWs focused on a specific disease, location, or prevention activity. Some CHWs delivered services primarily in the community while others were based in a clinical setting. Roles and responsibilities described by respondents were also broad. Some CHWs assisted patients in accessing clinical services, others supported patients' psycho-social needs, or served as a “cultural translator” between the patient and the health system. Some CHW roles required specific certifications such as HIV testing/counseling. CHWs acknowledged the breadth of their roles and responsibilities. One CHW stated, “We all do more than what we should do. We do a lot more than what we should be doing [CHW].” While another described a CHW’s role by saying, “They wear a lot of hats [CHW].” Respondents commonly reported that a CHW’s roles were not well understood. One administrator noted that when “trying to plug [in] community health workers, you’ll run into [this] kind of thing where nobody’s quite sure where they’re supposed to fit [administrator].” Also, respondents described a lack of clarity regarding what CHWs were responsible for and how responsibilities were divided within the team.

**Table 2:** Elements of a CHW’s roles and responsibilities

Category	Descriptions Used by Interview Respondents or Documents
Job Titles	**Formal HR job title**: Clinical Care Coordinator, Behavioral Health Coordinator, Program Service Aid, Community Affairs Specialist. **Informal team-level job title**: Community Health Worker, Outreach Worker, or Case Manager.
Target Population	**Disease-focus**: People with uncontrolled diabetes, people who inject drugs, HIV positive patients **Location-based**: Inpatient hospital, school-based health center **Health promotion or risk reduction-focus**: Needle exchange, oral health
Service Delivery Model	**Clinical Setting**: Doctors office, hospital **Community Setting**: Home, community-based organizations **Engagement model**: In person, phone, or telehealth
Roles	Health education, motivational interviewing, care coordination, case management, counseling, or community outreach.
Responsibilities	**Promoting access to clinical services**: Appointment scheduling, clinical intake, transportation, addressing barriers to care **Health service support**: Assisting with medication refills, health education **Psycho-social needs**: Supporting patients in obtaining jobs, housing, or personal identification; social service referrals; health insurance enrollment **Direct service**: Provision of food, toiletries, or clothing **Translation**: Language translation, helping providers understand patient experience, helping patients understand instructions from health care providers **Research:** Research study recruitment, enrollment, data collection **Documentation:** Data collection or entry for documentation purposes

#### Purpose/value

Respondents were asked why CHWs were employed as part of the team (the CHW’s purpose) and how the CHW contributed to helping patients or the team (their value). CHWs were perceived to play a critical role as “connectors” by serving as the linkage or middleman between the patient and the health system. Other respondents valued CHWs for their ability to build trusting relationships with patients. For some respondents, building trust required that CHWs invest time working closely with patients and the communities where they reside. But some discrepancy existed in how a CHW’s value was perceived among members of the team. Generally, CHWs emphasized their ability to reach or impact individual patients, framing their value from the perspective of the patient’s experiences or needs. While administrators and clinicians more commonly perceived a CHWs value in their ability to contribution to health service goals. For example, a CHW was valued by health providers or administrators for helping patients access care, improving physician efficiency, or reducing healthcare provider burnout. While all interviewed respondents valued CHWs and understood their purpose, respondents indicated that not all care team members shared this understanding (Table 3).

**Table 3:** Perceptions regarding a CHW’s role purpose, value, and effectiveness from the perspective of administrators/clinicians and CHWs

	Administrators or Clinicians	CHWs
Roles and Responsibilities	• **Supporting patients in accessing health services**^a^ • **Addressing psycho-social needs** • **Research study enrollment** • **Helping providers understand the lived experiences of patients**	• **Supporting patients in accessing health services** • **Addressing psycho-social needs** • **Research study enrollment** • **Helping providers understand the lived experiences of patients** • Helping patients understand instructions from health care providers • Recordkeeping
Purpose and Value	• **Building patient capacity to navigate health system** • **Improved health outcomes** • Facilitating access to care • Improved medication adherence • Reduced physician burnout • Reduced healthcare costs	• **Building patient capacity to navigate health system** • **Improving health outcomes** • Building relationships with patients • Helping patients feel valued • Working in the service of others • Making a difference in patient’s lives • Helping patients with complex psycho-social needs
Metrics of success	• **Patient engagement** • **Patient experience** • Return on Investment • No-show rates • Changes in disease metrics • Changes in health service utilization • Number of calls or visits completed	• **Patient engagement** • **Patient experience** • Trusting relationships with patients • Resourcefulness in accessing services • Success stories/direct feedback from patients • Feeling that they helped

^a^overlap/alignment bolded

One CHW noted, “It was where, you know, you’re *just* a community health worker... you’re just … you’re just … because they didn’t understand the work that we did and how valuable the work that we do is to the overall care of the patient.” Thus, there was a sense that understanding a CHW’s value was critical for effective integration. Some felt that the team’s leadership played an important role in articulating this value. One clinician noted, you “just really [need to] make sure that the leadership … understands the value and the importance of a community worker.”

#### Metrics of success

Respondents were asked how CHWs were evaluated and how they assessed whether CHWs were effective. While some common elements of evaluation emerged, there remained considerable diversity in evaluation metrics both across sub-units and between CHWs and administrators. Some CHWs were assessed on activities completed (number of calls or home visits completed), patient engagement (patient no-show rates), or biological metrics (hemoglobin A1C levels). Common evaluation tools included patient health assessments, patient satisfaction surveys, CHW activity reports, health action plans, or treatment plans. Some sub-units also tracked costs associated with CHW services to assess cost effectiveness or return on investment (ROI), others used healthcare utilization metrics (such as hospital readmission rates) to estimate a CHW’s impact. But perspectives on health and cost metrics were mixed. While these metrics were perceived to be valuable in sustaining funding and leadership support for CHW models, they were also perceived to be limited in their ability to properly capture a CHW’s value. Common was the perception that it takes time for CHWs to change individual health outcomes. One administrator noted, “It becomes very difficult for anyone to find money because then what they get used to doing is looking at what’s the direct return on investment immediately for this work. And, you know … it isn’t immediate …. and so the benefits might be a couple years down the road or even further [Administrator].” Consequently, a CHW’s positive impact may be missed by short-term evaluation cycles.

Additionally, CHW respondents reported that the full scope of their work was not properly captured by quantitative assessments of activities completed. CHWs described taking hours, weeks, or even months to build trust with patients to work toward health improvement goals. One CHW noted, “So, I do all these other little things that I don’t necessarily put on the chart. So yes, I do spend two hours or three hours with a client or however long it takes with the client because I need to make sure that the client is well taken care of and not just, you know, not just another number [CHW].” Assessments that relied heavily on numerical counts of CHW activities did not always capture the nuanced work essential for patients with complex health and psychosocial needs.

In addition to these formal metrics, CHWs commonly assessed their effectiveness based on qualitative experiences with patients. CHWs described visual assessments of patients either via observations in the home or during patient interactions. For example, one CHW observed patients for signs of recent drug use (fresh IDU tracks) while another observed child tooth brushing to determine the regularity of practice. CHWs also received direct feedback from patients via success stories, and they identified this direct feedback as critical in assessing effectiveness.

**Fig. 2:**
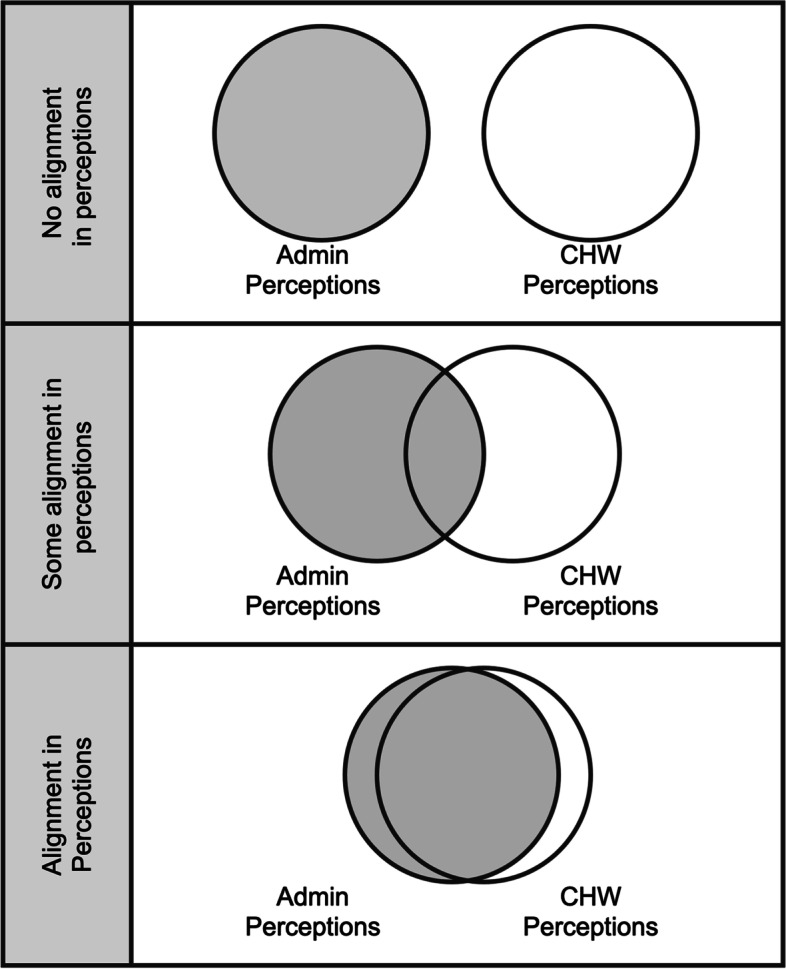
Relative alignment in perceptions of a CHWs role and purpose between groups

Thus, respondents reported inconsistent perceptions related to CHW roles and purpose. CHW roles were not always understood, and the CHW’s purpose and value were sometimes framed differently by different members of the care team. Moreover, evaluation metrics did not always effectively capture a CHW’s impact on the health care system. But in some cases, care teams were more aligned around a shared understanding of the CHW’s role and purpose within the care team (Fig. 2)—with some sub-units reporting little alignment and others reporting close alignment in perceptions.

### Integration

Respondents were asked how CHWs were integrated into the care team, how team members worked with CHWs, how CHWs and care team members communicated, and to what extent CHWs were integrated into care. Respondents reported a range of models for integration. Some sub-units employed CHWs to work directly with health care providers or care teams—sometimes in the same clinic or facility. While others facilitated integration of CHWs into care teams through regular check-ins such as huddles or meetings. And some CHWs had low levels of integration with care teams, working minimally with healthcare providers. Generally, a spectrum of integration was observed in which high clinical integration was associated with cohesive co-working among multidisciplinary members of the care team supported by clear information sharing channels (e.g. meetings and shared work space), systems that support co-working (e.g. clear structures and process), and a common goal (clear roles, training, and leadership support) and low levels of integration was associated with low or infrequent adherence with the critical integration factors described above. To assess integration, each sub-unit was scored for the presence/absence of 9 health-system and 7 community-level factors (Table 4). The process of evaluating CHWs along an integration spectrum has been described in another article [19].

**Table 4:** Factors assessed to evaluate integration of CHWs within clinical care teams

Health System Factors	Respondents reported working as part of care team Mechanisms for CHWs and care team members to communicate CHWs participated in regular meetings with care team CHWs had access to EMRs or other medical record systems CHW working in close proximity to care team members (share physical workspace) A champion or leader within the team supports CHWs integration A flattened hierarchy enables CHWs to engage in aspects of care Health care providers received training or mentorship in working with CHWs Protocols or procedures involve CHWs in health service delivery
Community Factors	Respondents reported integration with the communities served CHWs have shared experiences with patients or intimate knowledge of communities served CHWs work with patients where they live in homes or community settings close to patients CHWs have time to build relationships/rapport with patients CHWs are perceived as trusted members of the community Health services are delivered in a way that is easily accessed by patients Strong partnerships with other community organizations are maintained

Among subunits with high levels of CHW integration, alignment in perceptions regarding a CHW’s role and purpose was perceived to be critical for integration. When perceptions about CHWs were both positive and aligned, respondents reported higher levels of integration within the healthcare system. Thus, establishing positive CHW perceptions, via clear roles & responsibilities and purpose & value, supported CHW integration.

### Limitations

As a case study, this research focused specifically on one health and hospital system, and thus generalizability may be limited. Efforts were made to identify a case of study that shares traits with other health and hospital systems to improve generalizability. Additionally, by including only those programs willing to discuss CHW integration, recruitment practices may have selected for those programs with the most robust CHW integration models. But the relatively high response rate among recruited sub-units serves to minimize bias. It is also possible that biases may exist among respondents towards those individuals or teams who are more comfortable with organizational change or non-traditional care models. Or participants may be more likely to value a CHW compared to other care teams. Consequently, additional barriers may exist for those programs seeking to initiate CHW integration for the first time in a health system unaccustomed to CHW models.

## Discussion

This research highlights the importance of understanding how CHWs are perceived by different members of the care team. Programs with higher levels of integration had more alignment across administrators, clinicians and CHWs in the perceptions of a CHW’s purpose and value. Thus, this research suggests that clearly delineating expectations regarding a CHW’s role and purpose is a critical consideration in programs seeking to engage CHWs as part of clinical care teams. This finding aligns with other research which suggests that an important component of CHW effectiveness is clarity and alignment of expectations among members of the care team [12, 13, 31]. Alignment is of particular importance because of its ability to unite the team around a shared vision for the CHW model. But simply writing a clear job description is not sufficient. A CHWs role and purpose must also be articulated to members of the care team, and closely aligned with the metrics that are used to evaluate CHW effectiveness.

Furthermore, in healthcare settings, it is important to consider hierarchical and power dynamics that may influence CHW integration. Other research indicates that CHW’s may be particularly undervalued in clinical environments where “hard skills” such technical knowledge and educational credentials may be valued over “soft skills” such as communication, adaptability, and empathy [7, 23]. Thus, CHWs may be de-valued in comparison to more credentialed staff. Those in clinical and leadership positions must not only understand the purpose and value of CHWs, but they must also consider the unique challenges in employing CHWs within clinical care environments. Without leadership buy-in and support, CHWs risk facing critical challenges working in an environment that doesn’t understand what they do or how to work with them. Additionally, it is important that other members of the care team, including support staff, understand a CHW’s role. This is particularly important in ensuring that the care team understand that CHWs aren’t competing for responsibilities or resources.

One way in which CHW’s purpose and value can be demonstrated is with metrics that properly evaluate a CHW’s contribution in a healthcare setting. An overemphasis on short-term quantitative performance metrics may miss the complex and long-term impact of CHW models, and standard tools for evaluating health care effectiveness may not capture the impact of CHWs. Without clear, common evaluation measures that effectively capture a CHW’s value in a clinical care environment, it may be difficult to document and demonstrate a CHW’s purpose and value. Recent collaboratives have been working establish standardized process and outcome measures for CHW programs and interventions, thus contributing to alignment in metrics for CHW evaluation [26, 32]. This research suggests that work to identify standard and effective evaluation metrics may play an important role in aligning perceptions of CHWs which could improve CHW integration within health systems.

The field of systems thinking is growing in prominence within healthcare and public health sectors as practitioners seek to understand the complex adaptive context in which they are working [33]. While systems thinking itself is a broad transdiscipline marked by differing theoretical approaches, one commonly shared concept is the feedback loop. Feedback loops are based on the principle that causal pathways are not linear. They loop back on themselves in continuing cycles that result in exacerbating (reinforcing) or balancing ongoing change [20]. In feedback loops, effects have the potential to be compounded over time via either virtuous or vicious cycles [20]. The positive association between aligned perceptions and integration suggests the presence of a reinforcing feedback loop between perceptions and integration. The presence of a feedback loop has important considerations for CHW integration due to its potential to compound change. It suggests that even modest improvements in alignment around perceptions may contribute to substantial improvements in both alignment and integration over time. Thus, prioritizing alignment in perceptions is a critical component of CHW integration within healthcare teams.

Practitioners can use these findings to inform how they design and implement CHW programs within clinical care settings. This research highlights that prioritizing alignment in perceptions across all members of the clinical care team is a critical step in effectively integrating CHWs. It also provides additional support for broader efforts to align national perspectives on CHW roles, responsibilities, and evaluation metrics. But more questions remain regarding the best strategies to ensure alignment in perspectives across diverse care teams—especially among teams without previous knowledge of or experience with CHWs. More implementation research is needed to understand the best strategies for achieving alignment in perspectives regarding a CHW’s role, purpose, and value in a clinical care setting.

## Conclusion

A key finding of this research is the importance of alignment across a care team regarding a CHW’s role and purpose. This research also suggests that alignment and integration may be closely connected via a feedback loop. Thus, the goal of alignment isn’t one that is met, rather it is one that is refined. Often programs approach alignment by articulating the organization’s mission and ensuring that this mission aligns with the CHW model or by clearly drafting job responsibilities. While these steps are valuable in a movement toward alignment, it cannot be viewed as a “one and done” effort. Finding alignment reflects an ongoing process across multiple factors including the organization’s mission, the training and orientation process, the organization’s protocols and procedures, and amplifying CHW voices. Each step has the potential to contribute to ongoing improvements in both alignment and integration. Thus, leaders within care teams must ask what steps can be taken to improve alignment on an ongoing basis. Additionally, this research suggests that it is time for CHW programs to consider new tools for evaluating a CHW’s contribution. This research offers some insight into opportunities for innovation in evaluation. Qualitative assessments of CHW, provider, and patient experiences can give more nuanced insight. Additionally, lengthening the time horizon in which CHWs are evaluated could also serve to capture health improvements that take a longer time to realize. Finally, programs could consider using evaluation metrics for health system efficiency rather than patient outcomes.

## Supplementary Information

**Additional file 1.** Interview guide.

## Data Availability

The datasets used and/or analysed during the current study are available from the corresponding author on reasonable request.

## Abbreviations

CHW: Community Health Worker
UI Health: University of Illinois at Chicago Hospital and Health Sciences System
US: United States
ROI: Return on Investment
IDU: Intravenous Drug Use
