# Supplementary material for: Understanding the relationship between care team perceptions about CHWs and CHW integration within a US health system, a qualitative descriptive multiple embedded case study
Source: BMC Health Serv Res. 2022 Dec 27;22:1587. doi: 10.1186/s12913-022-08723-7 (PMC9793519; doi:10.1186/s12913-022-08723-7)
Supplement: Supplementary file 1 — Additional file 1. Interview guide. [file 12913_2022_8723_MOESM1_ESM.zip › Administrator Interview Guide_deidentified R2.docx]

**Administrator Interview Guide**

*Hi! My name is xx. I am a student at xx. Thank you for taking the time to meet with me. I am interested in speaking with you to learn more about your personal experiences working with Community Health Workers (CHW) within the UI Health System. I hope to use your experiences to better understand the opportunities and challenges for Community Health Workers at UIC. This may help to inform the development of a new UIC center that will serve to support CHWs across the University. I expect this interview to last between 60-90 minutes. I will be using this interview as part of my dissertation for xx. I will therefore be recording and transcribing this interview. To start, I wanted to share the results of a publicly available CHW report which was recently completed at UIC. Here is a copy of that report. You’re welcome to take a look at this before we start the interview.*

**Role/Responsibility**

1. Can you please describe your current role at UIC?
   1. What is your official job title? Do you have a different informal title?
   2. What are your primary roles and responsibilities?
   3. What department do you work in?
2. Can you please describe the ways in which you work with CHWs?
3. How long have you been working with CHWs?
4. Can you walk me through how CHWs are hired, trained and supervised?
   1. Who is responsible for hiring them? How does this work? [*Probes: what job codes are used? Who conducts the interviews?]*
   2. Who trains the CHW? What does training look like?
   3. How is the CHW supervised? [Probe: *If there are different supervisors, how do they work together?]* Who supervises the CHW managers?
   4. How are these procedures document (if at all)? [*Probe: are there written protocols?*]

**Clinical Care Teams**

*This section is applicable for clinicians who work in a clinical capacity at UIC.*

1. Do CHWs ever work with you in the provision of clinical care as part of a clinical care team? [*if no, skip to question 9]*
2. Can you please describe in what ways in which the CHW works with the clinical care team?
   1. Can you describe the culture of the clinical care team?
   2. How does the team communicate with the CHW?
   3. Do you ever work in a shared or common space with the CHW? If so, can you describe it to me?
   4. Are there any protocols or workflows that describe how to work with the CHW?
3. How well do you feel CHWs are integrated into (or part of) the care team? [*Probe: In what ways do you support the CHWs in their work? In what ways do they help you?]*

**Organization**

1. Can you tell me a little bit about what it is like to employ CHWs in the UI Health system?
2. How well do you think CHWs are integrated into the UI Health System? Why?
3. Has the organization needed to change at all in order to work with CHWs? If so how? What was that change process like?
4. In what ways does the organization support a CHW or CHW program?
5. How is your CHW/CHW program supported financially? How do you feel about the current system of financial support? [*potential probe*: *Is there anything that you would change?*]
6. Are leaders of your department or program involved in supporting CHWs (or the CHW program)? In what ways? How has this contributed to or hindered the CHWs.
7. How do CHWs support (or not support) the goals of your program/department?

**Objectives**

1. Why are you working with CHWs?
2. What are the primary jobs that the CHW performs?
3. How do you determine which patients a CHW should work with?
4. Do you think the CHW’s role is clearly defined and/or understood? Can you explain?
5. What do you value most about CHWs? [*probe: what problems do CHWs help to solve?*]
6. What do you think patients/clients value most about CHWs?
7. What are your goals/objectives for CHWs? When you think of CHWs why are they important to your work?
8. How do you (or your department) evaluate CHWs?
9. From your perspective, are CHWs meeting your goals/objectives?

**Gaps/Opportunities**

1. Can you please tell me a little bit about some of the biggest challenges or barriers to employing and/or working with CHWs?
   1. Do you have any challenges working as part of clinical care teams? What are they?
   2. Do you have any challenges working within the UI Health system? What are they?
2. What do you think is working really when it comes to CHWs?
3. How do you think we could build upon those things that are working well?
4. In what ways do you think your program and CHWs in general could be supported? What do you need?
   1. *Possible addition*: Here are the results from the survey? How do you feel about these? Do any of them resonate for you? Is there anything missing?
5. If there was a CHW Center at UIC, what would you want it to do?
