# Supplementary material for: Understanding the relationship between care team perceptions about CHWs and CHW integration within a US health system, a qualitative descriptive multiple embedded case study
Source: BMC Health Serv Res. 2022 Dec 27;22:1587. doi: 10.1186/s12913-022-08723-7 (PMC9793519; doi:10.1186/s12913-022-08723-7)
Supplement: Supplementary file 1 — Additional file 1. Interview guide. [file 12913_2022_8723_MOESM1_ESM.zip › CHW Interview Guide_deidentifiedR2.docx]

**CHW Interview Guide**

*Hi! My name is xx. I am a student at the xx. Thank you for taking the time to meet with me. I am interested in speaking with you to learn more about your personal experiences working as a Community Health Worker (CHW) within the UI Health System. I hope to use your experiences to better understand the opportunities and challenges for Community Health Workers at UIC. This may help to inform the development of a new UIC center that will serve to support CHWs across the University. I expect this interview to last between 60-90 minutes. I will be using this interview as part of my dissertation for xx. I will therefore be recording and transcribing this interview. To start, I wanted to share the results of a publicly available CHW report which was recently completed at UIC. Here is a copy of that report. You’re welcome to take a look at this before we start the interview.*

**Role/Responsibility**

1. Can you please describe your current role at UIC?
   1. What is your official job title? Do you have a different informal title?
   2. What are your primary roles and responsibilities? How clear are these responsibilities? Do they ever change?
   3. How long have you worked in your role?
   4. What department do you work in?
   5. Who do you report to? How are you supervised?
2. What do you view your purpose to be in your job?
3. Can you please tell me a little bit about your training?

**Clinical Care Teams**

1. Do you work with any clinical care providers or a clinical care team? [*if no, skip to question xx]*
2. How are you assigned patients to work with?
3. Can you please describe in what ways you work with clinical providers or a clinical care team? Maybe you can walk me through a typical day?
   1. How does the team communicate with each other? How do they communicate with you?
   2. Do you ever work in a shared or common space with the clinical team? If so, can you describe it to me?
   3. Are there any protocols or workflows that describe how you and the care team works together?
4. How well do you feel you are integrated, engaged or included into (or part of) the care team? [*Probe: In what ways do you support the care team in their work? In what ways do they help you?]*
   1. Can you describe some of the things that help you work well with the clinical care team?
   2. Can you describe some things that make it difficult for you to work with the care team?

**Organization**

1. Do you feel that UIC as an organization values you as an employee? Why/why not?
2. How well do you think you are integrated into the UI Health System?

**Community**

1. Can you describe to me how you work in a community or neighborhood outside of UIC (if at all)? What do you do?
2. How do you define the community that you work with?
3. How much time would you say that you work in a community setting?
4. How do you feel you are perceived by the community?
5. How do you feel that UIC is viewed by the community?
6. How do you feel about the balance of your time between the community and the clinic? Do you with you spent your time differently?
7. What are some of your primary challenges when working in the community?

**Objectives**

1. What do you think your patients/clients value most about you?
2. What do you think the people that you work with at UIC value most about you?
3. In what ways do you contribute to the work being done by clinicians at UIC?
4. In what ways do you contribute to the health of the patients/clients that you work with?
5. How do you track whether your patients have made progress? How do you know when they no longer need services?
6. Do you receive feedback on your work? Who gives you this feedback and how is it shared with you? How often?
7. What are your own personal goals in your work?
8. How successful are you in meeting your goals?
9. What do you like most about your job? How much time do you spend doing this part of your job?
10. Is there anything you would change about your job? What is it? Why?

**Gaps/Opportunities**

1. Can you please tell me a little bit about some of your biggest challenges or barriers?
2. What do you think is working really well in your current job?
3. How do you think we could build upon those things that are working well?
4. In what ways do you think you could be supported better? What do you need?
   1. *Possible addition*: Here are the results from the survey? How do you feel about these? Do any of them resonate for you? Is there anything missing?
5. If there was a CHW Center at UIC, what would you want it to do?
